# Atrial fibrillation type-specific prediction of recurrence after catheter ablation: the pivotal role of right atrial remodeling revealed by explainable machine learning

**DOI:** 10.3389/fcvm.2026.1805262

**Published:** 2026-04-29

**Authors:** Xiaonan Han, Tianyun Liu, Meng Li, Yirong Liu, Fang Li, Tong Pan, Dongyan Liu

**Affiliations:** 1 School of Medical Imaging, Hebei Medical University, Shijiazhuang, Hebei, China; 2 Department of Radiology, Hebei General Hospital, Shijiazhuang, Hebei, China; 3 Hebei Key Laboratory of Medical Imaging, Hebei Medical University, Shijiazhuang, Hebei, China

**Keywords:** atrial fibrillation, catheter ablation, explainable artificial intelligence, machine learning, recurrence prediction, right atrium

## Abstract

**Background:**

The high recurrence rate following catheter ablation for atrial fibrillation (AF) remains a significant clinical challenge. Existing prediction models are predominantly limited to left atrial parameters and often fail to distinguish between AF types, resulting in suboptimal predictive accuracy and clinical utility. This study aimed to develop distinct machine learning (ML) models for predicting recurrence in patients with paroxysmal AF (PaAF) and persistent AF (PeAF), with a specific focus on evaluating the predictive value of right atrial structural parameters. Furthermore, explainable artificial intelligence (XAI) techniques were employed to decipher the decision-making mechanisms of the models.

**Methods:**

We retrospectively enrolled 297 patients who underwent radiofrequency catheter ablation (RFCA) for AF (230 in the PaAF group; 67 in the PeAF group). A total of 37 clinical and cardiac computed tomography (CT) imaging features were collected. Following feature selection, eight ML algorithms were trained and evaluated. The SHapley Additive exPlanations (SHAP) framework was used to provide model interpretability, and a clinical decision support tool was developed.

**Results:**

The XGBoost and LightGBM models demonstrated superior predictive performance for PaAF [Area Under the Receiver Operating Characteristic Curve (AUC): 0.831] and PeAF (AUC: 0.917), respectively. SHAP analysis identified the right atrial appendage (RAA) short diameter as the most important predictor for PaAF recurrence, whereas right atrial (RA) volume was the top contributor to predicting PeAF recurrence. Feature dependence plots further revealed complex nonlinear relationships and interaction effects.

**Conclusion:**

Machine learning models exhibited excellent performance in predicting post-ablation AF recurrence, with right atrial structural parameters emerging as key predictors. The explainable framework and clinical decision support tool developed in this study provide a new paradigm for precise prognosis assessment and personalized management following AF ablation.

## 1 Introduction

Atrial fibrillation (AF) is one of the most common clinical arrhythmias, and its rising global prevalence contributes to complications such as stroke and heart failure, imposing a substantial socioeconomic burden (1, 2). Notably, modifiable lifestyle factors such as smoking have been established as causal risk factors for AF, underscoring the importance of prevention (3). For drug-refractory atrial fibrillation, radiofrequency catheter ablation (RFCA) has become a first-line therapeutic strategy. However, post-procedural AF recurrence rates remain high, with long-term follow-up studies indicating recurrence rates of 10%–30% (4). Therefore, accurately identifying patients at high risk of recurrence is crucial for managing post-procedural expectations and formulating individualized follow-up and intervention strategies.

The pathophysiological substrate of AF is characterized by complex and progressive structural remodeling. Histological studies have demonstrated that this substrate extends beyond interstitial fibrosis to include cardiomyocyte interstitial space enlargement, myofibril loss, and a reduction in cardiomyocyte nuclear density, collectively contributing to the electrophysiological abnormalities seen in AF (5). This remodeling process affects both atria, with increasing evidence highlighting the significant role of the right atrium (RA) in AF initiation and maintenance. Imaging studies, such as those using late gadolinium enhancement cardiac magnetic resonance, have shown that RA structural and functional remodeling occurs in parallel with left atrial (LA) changes and that specific RA parameters, like RA sphericity, can serve as independent predictors of ablation outcomes (6).

Various clinical risk scores (e.g., APPLE, CAAP-AF) and prediction models based on traditional statistical methods (e.g., Cox regression) have been proposed to predict post-ablation recurrence (7, 8). However, the discriminatory power of these models is often unstable across different cohorts, limiting their widespread clinical application (9). Key limitations include: (1) the difficulty of traditional methods in capturing complex nonlinear relationships and interaction effects among variables; (2) a reliance on a limited set of clinical features, failing to fully integrate multimodal data, particularly detailed cardiac imaging parameters; (3) a predominant focus on left atrial parameters, with insufficient consideration of the right atrium, despite growing evidence of its prognostic importance, and (4) the common practice of combining paroxysmal AF (PaAF) and persistent AF (PeAF) despite their potential differences in pathophysiological substrate and recurrence mechanisms.

In recent years, machine learning has shown significant advantages in medical predictive modeling by automatically learning complex mappings between high-dimensional features and outcomes. This capability allows it to outperform traditional models in predicting AF recurrence after ablation (10–12). However, ML models are often perceived as “black boxes” due to their opaque decision-making processes, hindering clinical trust and adoption. Explainable artificial intelligence (XAI) techniques, such as SHapley Additive exPlanations (SHAP), address this by quantifying feature contributions to predictions, providing both global and local model explanations. Consequently, XAI enhances model transparency and boosts clinical acceptability (13, 14).

Despite these advances, critical gaps remain. First, most studies have not systematically developed separate prediction models for PaAF and PeAF, which exhibit fundamental differences in atrial substrate remodeling. Consequently, their recurrence mechanisms and predictors may differ (15). Second, existing models predominantly focus on left atrial and pulmonary vein imaging features, with insufficient attention paid to right atrial structural parameters. Growing evidence suggests the right atrium plays a significant role in AF initiation and maintenance, and its structural and functional changes may be associated with ablation outcomes (16, 17). For instance, a recent CT imaging study by Pan et al. (18) found that the right atrial appendage (RAA) short diameter was an independent predictor for PaAF recurrence, while RAA height and crista terminalis thickness were more critical for PeAF recurrence. This highlights the potential of incorporating right atrial parameters to improve prediction accuracy.

Therefore, systematically evaluating the value of right atrial parameters in stratifying recurrence risk across AF types using ML and elucidating their mechanisms is highly important. Based on this background, our study aims to: (1) develop and validate separate ML models for predicting recurrence after RFCA in PaAF and PeAF patients, systematically comparing multiple algorithms; (2) comprehensively incorporate cardiac computed tomography (CT) features, focusing on the predictive value of detailed right atrial and RAA morphological parameters; (3) apply the SHAP framework to identify key predictors, elucidate their direction of effect and nonlinear relationships, providing new insights into recurrence mechanisms; and (4) develop a prototype web-based clinical decision support tool that integrates high-performance prediction with model explainability, facilitating the translation towards personalized AF management.

## 2 Methods

### 2.1 Study design and patient population

This single-center, retrospective study initially enrolled 326 patients who underwent their first RFCA for AF with pre-procedural 256-slice spiral CT scans between January and October 2020. AF diagnosis was confirmed by clinical electrocardiogram and physical examination. Patients were categorized into PaAF (*n* = 230), defined as self-terminating episodes lasting ≤7 days (typically <48 h), and PeAF (*n* = 67), defined as continuous AF sustained beyond 7 days requiring cardioversion for termination.

Inclusion criteria comprised: (1) patients undergoing their first successful RFCA procedure; (2) availability of pre-procedural 256-slice CT angiography; (3) absence of major periprocedural complications (e.g., cardiac tamponade, atrio-esophageal fistula, periprocedural myocardial infarction, stroke, or pericardial hemorrhage); and (4) complete follow-up data. Exclusion criteria were: (1) poor image quality in CT angiography affecting measurements of RAA, RA volume, or crista terminalis thickness; (2) contraindications to CT angiography examination (e.g., contrast allergy, pacemaker); (3) significant structural heart disease. Ultimately, 29 patients were excluded due to inadequate image quality, resulting in a final cohort of 297 patients. The study was approved by the hospital ethics committee (NO.2018-R245), with written informed consent obtained from all participants.

### 2.2 Follow-up and endpoint definition

Patients were followed up via telephone or outpatient clinic visits. AF recurrence was diagnosed based on surface electrocardiogram or 24 h Holter monitoring. Recurrence was defined as any documented atrial tachyarrhythmia (atrial tachycardia, atrial flutter, or AF) lasting >30 s after a 3-month blanking period. Twelve-lead ECGs were performed at 3, 6, and 12 months post-ablation, with additional 24 h Holter monitoring at 6 and 12 months. Patients were encouraged to seek immediate medical attention or use portable ECG recorders if symptomatic.

### 2.3 Feature collection and selection

**Figure 1:**
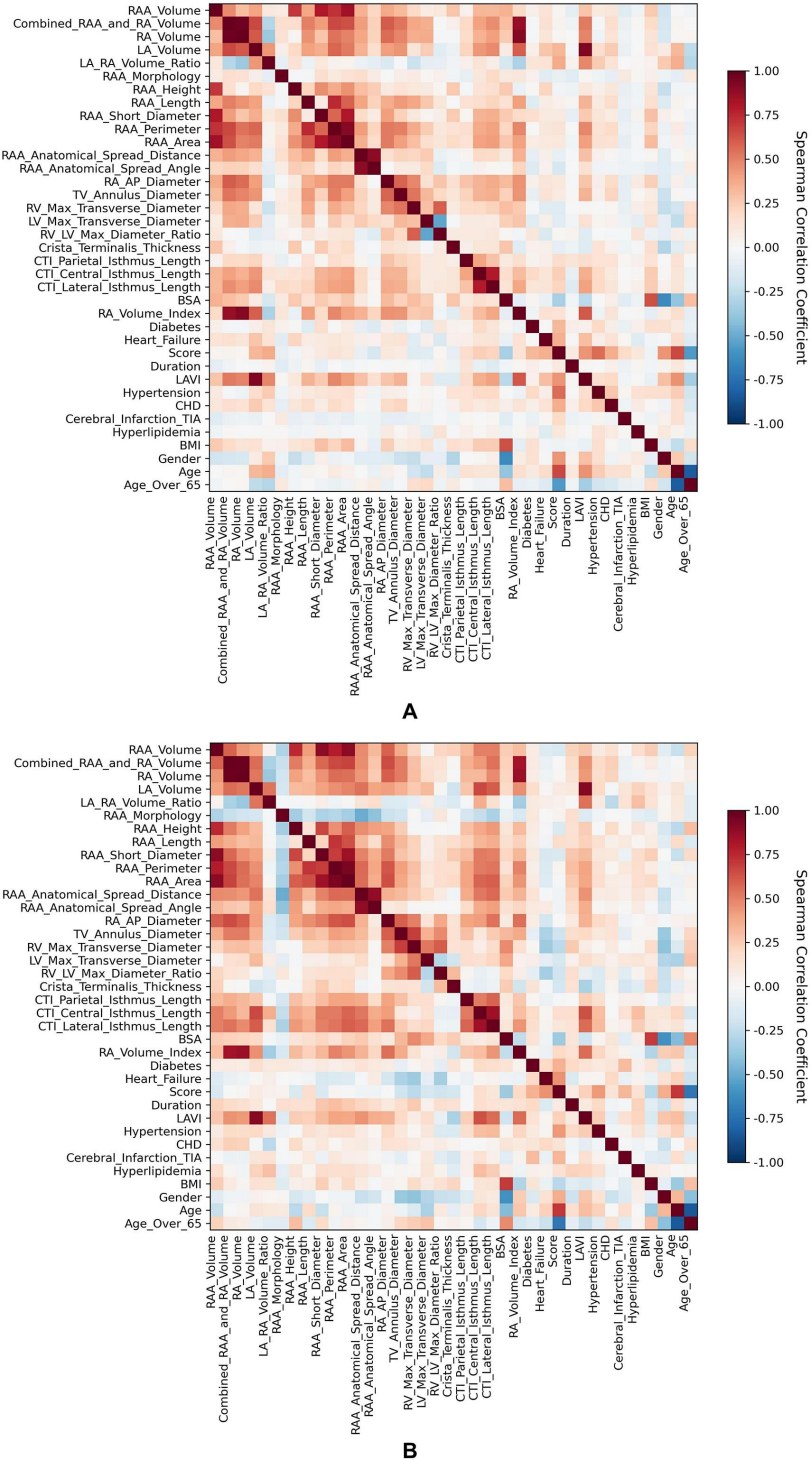
Spearman correlation heatmap of features in patients with **(A)** paroxysmal atrial fibrillation (PaAF) and **(B)** persistent atrial fibrillation (PeAF). The color intensity represents the strength of the correlation between features (red indicates positive correlation, blue indicates negative correlation). This analysis was used to identify and remove features with high collinearity (|correlation coefficient| >0.7) to optimize model input.

The dataset comprised 37 features spanning demographics, cardiac anatomy, and clinical comorbidities (Tables 1, 2). To mitigate multicollinearity, Spearman correlation analysis was first performed. The Spearman correlation heatmap of features is presented in Figure 1. Features with an absolute correlation coefficient >0.7 and lower importance based on Random Forest were considered redundant and removed. Given the potential for different predictors between AF types, feature selection was conducted separately for the PaAF and PeAF datasets using both the Boruta algorithm (a wrapper method around Random Forest) (19) and Recursive Feature Elimination with Cross-Validation (RFECV). The intersection of features selected by both algorithms was used as the final feature set, ensuring robustness. Should the intersection of features selected by Boruta and RFECV be empty, the feature set confirmed by the Boruta algorithm will be adopted as the final set for modeling. In such a case, the RFECV filtering step will be omitted, and a brief discussion regarding the potential reasons—pertaining to either the data characteristics or parameter settings—that led to this outcome will be provided to ensure methodological rigor.

**Table 1:** Comparison of baseline characteristics between recurrent and non-recurrent groups in patients with PaAF.

Category	Parameters	Non-recurrent (*n* = 177)	Recurrent (*n* = 53)	*P* value
Demographics	Gender(Males)	97 (54.80%)	30 (56.60%)	0.941
	Age(years)	61.00 (56.00, 67.00)	65.00 (57.00, 71.00)	0.083
	Age > 65	54 (30.51%)	24 (45.28%)	0.068
	BMI(kg/m^2^)	25.80 (23.73, 27.85)	24.98 (23.18, 27.64)	0.367
Cardiac Anatomy	BSA (m^2^)	1.78 ± 0.18	1.79 ± 0.19	0.779
	RAA Volume(mL)	8.30 (6.30, 10.90)	10.50 (8.90, 14.00)	<0.001
	RAA Morphology(L type)	33 (18.64%)	5 (9.43%)	0.099
	RAA Height(mm)	25.50 (22.00, 30.00)	28.40 (25.70, 33.00)	<0.001
	RAA Length(mm)	34.75 ± 5.55	34.94 ± 5.09	0.815
	RAA Short Diameter(mm)	22.16 ± 5.21	26.94 ± 5.07	<0.001
	RAA Perimeter(mm)	105.00 ± 13.58	112.30 ± 13.35	<0.001
	RAA Area (mm^2^)	652.62 (573.68, 778.40)	766.65 (658.58, 891.32)	<0.001
	RAA Anatomical Spread Distance(mm)	20.40 (15.10, 24.70)	22.70 (15.80, 26.80)	0.098
	RAA Anatomical Spread Angle (°)	15.70 (10.80, 21.10)	18.60 (12.50, 24.20)	0.105
	Combined RAA and RA Volume(mL)	77.90 (67.30, 93.70)	88.0 (72.10,102.90)	0.038
	RA Volume(mL)	69.70 (60.70, 83.70)	73.30 (59.10, 89.40)	0.279
	RA Volume Index	39.68 (34.35, 46.44)	41.60 (35.61, 49.31)	0.302
	RA AP Diameter(mm)	44.60 (40.90, 47.70)	46.30 (42.90, 48.60)	0.147
	LA Volume(mL)	88.00 (75.00, 104.90)	96.10 (77.40, 117.00)	0.061
	LA/RA Volume Ratio	1.21 (1.07, 1.42)	1.25 (1.10, 1.55)	0.219
	LAVI(mL/m^2^)	50.33 ± 13.19	57.08 ± 18.67	0.017
	TV Annulus Diameter(mm)	35.90 (32.40, 38.70)	37.00 (34.40, 39.00)	0.041
	RV Max Transverse Diameter(mm)	37.50 (34.20, 41.90)	37.40 (34.70, 41.40)	0.901
	LV Max Transverse Diameter(mm)	41.60 (38.80, 45.10)	40.60 (38.00, 43.80)	0.140
	RV/LV Max Diameter Ratio	0.89 (0.81, 0.97)	0.92 (0.84, 1.01)	0.301
	Crista Terminalis Thickness(mm)	3.40 (3.10, 3.90)	3.70 (3.40, 4.30)	<0.001
	CTI Parietal Isthmus Length(mm)	15.50 (14.60, 16.40)	15.70 (14.70, 16.40)	0.559
	CTI Central Isthmus Length(mm)	18.70 (16.90, 21.00)	20.50 (18.10, 23.00)	0.003
	CTI Lateral Isthmus Length(mm)	22.78 ± 3.42	24.41 ± 4.14	0.011
Clinical Comorbidities	Diabetes	25 (14.12%)	14 (26.42%)	0.060
	Hypertension	99 (55.93%)	28 (52.83%)	0.810
	Coronary Heart Disease	100 (56.50%)	32 (60.38%)	0.732
	Heart Failure	27 (15.25%)	13 (24.53%)	0.175
	Hyperlipidemia	41 (23.16%)	13 (24.53%)	0.983
	Cerebral Infarction TIA	29 (16.38%)	7 (13.21%)	0.732
	Duration(months)	12.00 (2.00, 48.00)	24.00 (6.00, 60.00)	0.011
	CHA2DS2-VASc Score	2.00 (1.00, 4.00)	3.00 (2.00, 4.00)	0.217

Data are presented as: continuous variables with normal distribution as mean ± standard deviation, non-normally distributed variables as median (interquartile range), and categorical variables as frequency (percentage). Intergroup comparisons: normally distributed continuous variables were analyzed using independent samples *t*-test, non-normally distributed continuous variables using Mann–Whitney *U* test, and categorical variables using Chi-square test or Fisher's exact test. *P* values are shown as numerical values, and a *P* < 0.05 was considered statistically significant. BMI, body mass index; BSA, body surface area; RAA, right atrial appendage; RA, right atrium; LA, left atrium; LAVI, left atrial volume index; TV, tricuspid valve; RV, right ventricle; LV, left ventricle; CTI, cavotricuspid isthmus; TIA, transient ischemic attack.

**Table 2:** Comparison of baseline characteristics between recurrent and non-recurrent groups in patients with persistent atrial fibrillation.

Category	Parameters	Non-recurrent (*n* = 37)	Recurrent (*n* = 30)	*P* value
Demographics	Gender (Males)	26 (70.27%)	20 (66.67%)	0.959
	Age (years)	63.00 (55.00, 68.00)	60.50 (57.00, 69.00)	0.840
	Age > 65	14 (37.84%)	11 (36.67%)	1.000
	BMI(kg/m^2^)	26.02 ± 3.34	25.80 ± 3.09	0.786
Cardiac Anatomy	BSA(m^2^)	1.83 ± 0.20	1.83 ± 0.18	0.952
	RAA Volume(mL)	8.70 (7.30, 12.20)	14.65 (13.60, 17.65)	<0.001
	RAA Morphology(L type)	5 (13.51%)	3 (10.00%)	0.676
	RAA Height(mm)	25.12 ± 5.38	30.84 ± 4.41	<0.001
	RAA Length(mm)	37.25 ± 5.06	39.29 ± 5.10	0.108
	RAA Short Diameter(mm)	21.90 (20.70, 25.70)	30.30 (27.15, 33.83)	<0.001
	RAA Perimeter(mm)	111.96 ± 12.64	126.85 ± 14.28	<0.001
	RAA Area(mm^2^)	771.20 ± 188.62	1,029.11 ± 233.03	<0.001
	RAA Anatomical Spread Distance(mm)	24.10 ± 6.99	28.02 ± 8.72	0.051
	RAA Anatomical Spread Angle (°)	18.54 ± 6.43	21.66 ± 7.53	0.077
	Combined RAA and RA Volume(mL)	102.40 (91.80, 111.00)	132.2 (112.33, 159.65)	<0.001
	RA Volume(mL)	91.40 (82.30, 100.70)	115.20 (99.33, 141.60)	<0.001
	RA Volume Index	51.33 ± 8.59	64.12 ± 19.05	0.002
	RA AP Diameter(mm)	48.20 (45.70, 49.40)	52.55 (47.00, 57.95)	0.007
	LA Volume(mL)	113.12 ± 25.82	139.77 ± 41.23	0.003
	LA/RA Volume Ratio	1.23 ± 0.29	1.23 ± 0.28	0.969
	LAVI(mL/m^2^)	58.39 (55.43, 73.26)	75.45 (58.22, 90.70)	0.011
	TV Annulus Diameter(mm)	38.10 (36.60, 43.10)	41.85 (38.20, 44.98)	0.060
	RV Max Transverse Diameter(mm)	40.83 ± 5.94	39.55 ± 7.68	0.458
	LV Max Transverse Diameter(mm)	44.53 ± 6.25	43.95 ± 7.43	0.734
	RV/LV Max Diameter Ratio	0.93 ± 0.14	0.91 ± 0.15	0.608
	CristaTerminalis Thickness(mm)	3.40 (3.10, 3.70)	3.80 (3.12, 4.90)	0.064
	CTI Parietal Isthmus Length(mm)	16.11 ± 1.57	17.37 ± 2.55	0.022
	CTI Central Isthmus Length(mm)	20.56 ± 3.25	23.62 ± 3.49	<0.001
	CTI Lateral Isthmus Length(mm)	24.84 ± 3.23	27.96 ± 4.63	0.003
Clinical Comorbidities	Diabetes	5 (13.51%)	7 (23.33%)	0.470
	Hypertension	16 (43.24%)	22 (73.33%)	0.716
	CHD	20 (54.05%)	16 (53.33%)	1.000
	Heart Failure	16 (43.24%)	15 (50.00%)	0.760
	Hyperlipidemia	7 (18.92%)	7 (23.33%)	0.889
	Cerebral Infarction TIA	9 (24.32%)	6 (20.00%)	0.899
	Duration(months)	24.00 (3.00, 36.00)	30.00 (2.00, 114.00)	0.149
	CHA2DS2-VASc Score	2.00 (2.00, 4.00)	3.00 (2.00, 4.00)	0.352

Data presentation and statistical methods are consistent with those in Table 1. Abbreviations are consistent with those in Table 1.

### 2.4 Data preprocessing

1. Categorical Encoding: all categorical features were converted into numerical format.
2. Outlier Handling: outliers in continuous features were identified and processed using the Interquartile Range (IQR) method.
3. Class Imbalance Handling: the PaAF group exhibited significant class imbalance (non-recurrent: 177, 77.0%; recurrent: 53, 23.0%). To address this and prevent model bias towards the majority class, the Adaptive Synthetic Sampling (ADASYN) technique was applied (20). Critically, to avoid data leakage, any synthetic samples generated by ADASYN were confined strictly to the training data. Specifically, the original PaAF training set comprised 161 patients (37 recurrent, 124 non-recurrent). Following ADASYN oversampling, the training set was expanded to 241 samples, resulting in a balanced distribution of 117 recurrent and 124 non-recurrent patients. All subsequent model training was conducted on this balanced training set. The final model evaluation was performed on a completely independent test set consisting solely of original, unseen patient data.
4. Standardization and Split: all continuous features were standardized using Z-scores. The data was then split into training and test sets (7:3 ratio) using stratified random sampling to maintain distribution stability.

### 2.5 Model construction and evaluation

Prediction was performed separately for PaAF and PeAF groups. Eight ML algorithms were compared: Logistic Regression, Support Vector Machine (SVM), K-Nearest Neighbors (KNN), Decision Tree, Random Forest, AdaBoost, XGBoost, and LightGBM. For each algorithm, hyperparameter optimization was performed using a grid search strategy with 5-fold cross-validation on the training set to maximize the AUC. The hyperparameter search spaces for key algorithms are detailed in Supplementary Tables S3, S4. The generalization ability of the optimal model from each algorithm was then evaluated on the held-out independent test set (30% of the total data). Performance metrics included AUC, accuracy, precision, recall, and F1-score. For each AF type, the algorithm that achieved the highest AUC on the test set was selected as the final optimal model for subsequent explainability analysis.

Given the relatively smaller sample size in the PeAF cohort, bootstrap validation was additionally performed for the optimal model to obtain a robust and stable estimate of its generalization performance (21, 22). Specifically, we employed the bootstrap method with 1,000 iterations. In each iteration, a bootstrap sample was generated by drawing, with replacement, a number of observations equal to the size of the original PeAF training set. The optimal model was retrained on this bootstrap sample and then evaluated on the independent hold-out test set (which remained strictly unseen during all resampling iterations) to calculate the AUC. This process, repeated 1,000 times, produced a distribution of AUC estimates. The median AUC from this distribution was reported as the performance estimate, accompanied by its 95% confidence interval derived from the percentile method. This approach mitigates the optimism associated with a single train-test split and provides a more reliable assessment of the model's expected performance on independent data by simulating the variability arising from different training samples.

### 2.6 Clinical utility assessment

To assess the practical clinical value of our predictive models beyond traditional discrimination metrics, we performed Decision Curve Analysis (DCA) (23, 24). Unlike the AUC, which evaluates overall model discrimination, DCA quantifies the net benefit of using a model to guide clinical decisions across a continuous range of probability thresholds. This method directly compares the net benefit of a model-based strategy against two default clinical strategies: intervening on all patients (“Treat All”) or intervening on none (“Treat None”). The net benefit for a given risk threshold (*p*) was calculated using the standard formula:

$$NetBenefit=\frac{TP_{p}}{N}-\frac{FP_{p}}{N}\times \frac{\;p}{1-p}$$

where *TP_p_* and *FP_p_* represent the number of true positives and false positives, respectively, when the model's predicted probability exceeds the threshold *p*, and *N* is the total number of patients in the test set. In practice, we evaluated 98 equally spaced threshold probabilities between 0.01 and 0.99. For each threshold, the net benefit was calculated for the optimal model and for the two reference strategies. The results were plotted with the risk threshold on the *x*-axis and the net benefit on the *y*-axis. A model is considered clinically useful within a range of thresholds where its decision curve lies above the curves for “Treat All” and “Treat None”, indicating that it adds more clinical value than the default approaches. This analysis was conducted for the optimal model in each AF subtype cohort.

### 2.7 Model interpretability analysis

To enhance clinical trust, the optimal models were interpreted using the SHAP framework. SHAP values quantify the contribution of each feature to individual predictions fairly and consistently. The global importance of a feature was calculated as the mean absolute SHAP value across all samples. The analysis included:

1. Global Interpretability:Using summary plots and feature importance rankings to identify the most important features and their direction of effect on recurrence risk.
2. Local Interpretability:Using waterfall plots to illustrate how each feature value contributes to shifting the predicted recurrence probability from the base value (average model output) for individual patients, providing transparent justification for personalized decisions.

## 3 Results

### 3.1 Patient baseline characteristics and feature selection results

Baseline characteristics for PaAF patients (recurrence rate 23%) are shown in Table 1. The recurrence group had significantly higher values for several key parameters, including RAA volume, height, short diameter, perimeter, area and crista terminalis thickness (all *P* < 0.001). Additionally, significant differences were also observed in LAVI (*P* = 0.017) and tricuspid valve annulus diameter (*P* = 0.041). Table 2 shows characteristics for PeAF patients (recurrence rate 45%). The recurrence group demonstrated significantly higher values for several key right atrial structural parameters (e.g., RAA volume, RAA short diameter, RA volume) compared to the non-recurrence group (all *P* < 0.001). No significant differences were found in gender, BMI, BSA, or comorbidities like hypertension and coronary disease between recurrence groups for both AF types.

In this study, since the feature sets selected by the Boruta and RFECV methods had a non-empty intersection, we adopted this common subset as the final feature set for model training. This process yielded 14 features for the PaAF group (e.g., RA volume, LA volume, RAA short diameter) and 9 features for the PeAF group (e.g., RA AP Diameter, RV Max Transverse Diameter, RA Volume). These final feature sets are listed in Table 3. Additionally, a comprehensive process of feature selection was provided in the Supplementary Table S1, S2.

**Table 3:** Final optimal feature sets selected for the PaAF and PeAF prediction models.

Class	Number of Features	Feature Set
PaAF	14	RA Volume, LA Volume, LA RA Volume Ratio, RAA Short Diameter, RAA Perimeter, TV Annulus Diameter, RV Max Transverse Diameter, RV LV Max Diameter Ratio, LV Max Transverse Diameter, Crista Terminalis Thickness, CTI Parietal Isthmus Length, CTI Central Isthmus Length, Duration, Age
PeAF	9	RA AP Diameter, RV Max Transverse Diameter, RA Volume, RV LV Max Diameter Ratio, Crista Terminalis Thickness, Duration, RAA Volume, CTI Lateral Isthmus Length, LAVI

The features listed represent the intersection derived from both the Boruta algorithm and recursive feature elimination with cross-validation.

### 3.2 Model development and performance comparison

A comparative analysis of eight machine learning models was conducted separately for the PaAF and PeAF cohorts. The evaluation on the independent test set revealed that the XGBoost and LightGBM algorithms achieved the highest predictive performance for the PaAF and PeAF groups, respectively. Therefore, these two models were selected as the optimal models for their respective AF types and were subjected to further explainability analysis and clinical utility assessment.

### 3.3 ROC curve analysis

**Figure 2:**
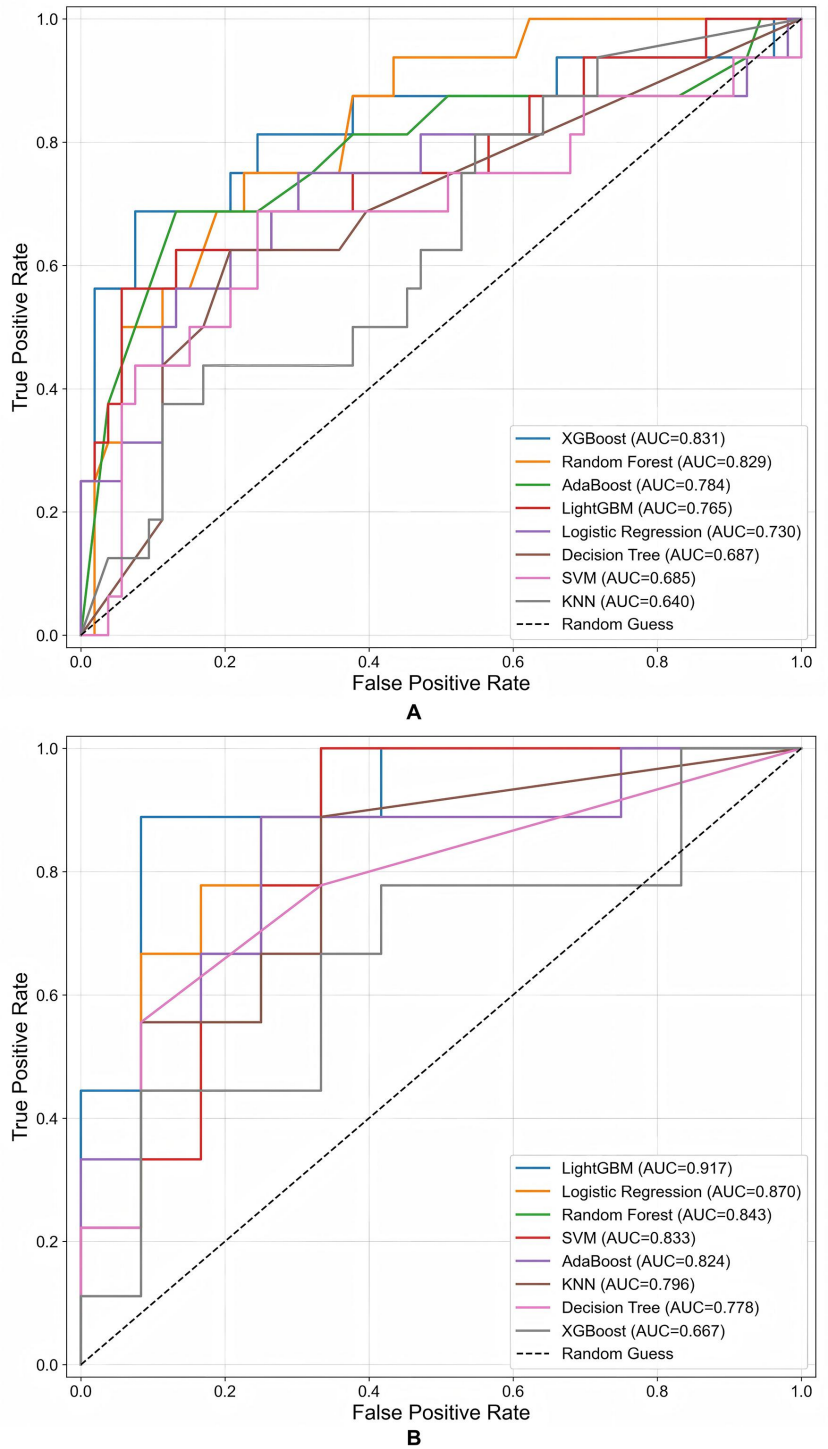
Receiver operating characteristic (ROC) curves of the eight machine learning models for predicting AF recurrence after ablation. **(A)** PaAF group: XGBoost showed the best performance (AUC = 0.831). **(B)** PeAF group: LightGBM performed best (AUC = 0.917).

The predictive performance of all models is comprehensively detailed in Table 4 (PaAF group) and Table 5 (PeAF group), with the corresponding ROC curves visualized in Figure 2. Consistent with the selection process outlined in Section 3.2, the XGBoost model demonstrated superior discriminative ability in the PaAF group, achieving an AUC of 0.831. Conversely, the LightGBM model excelled in the PeAF group with an AUC of 0.917. An overview of the ROC curves in Figure 2 confirms that ensemble methods, including XGBoost, LightGBM, and Random Forest, generally outperformed other algorithms across both cohorts.

**Table 4:** Performance comparison of eight machine learning models on the original, unmodified test set for PaAF. The best-performing results are shown in bold.

Model	AUC	Accuracy	Precision	Recall	F1-Score
XGBoost	**0** **.** **831**	**0**.**841**	**0**.**647**	**0**.**688**	**0**.**667**
Random Forest	0.829	0.826	0.700	0.438	0.538
AdaBoost	0.784	0.667	0.394	0.813	0.531
LightGBM	0.765	0.826	0.643	0.563	0.600
Logistic Regression	0.730	0.754	0.474	0.563	0.514
Decision Tree	0.687	0.783	0.538	0.438	0.483
SVM	0.685	0.786	0.500	0.500	0.500
KNN	0.640	0.594	0.286	0.500	0.364

**Table 5:** Performance comparison of eight machine learning models on the test set for PeAF. The best-performing results are shown in bold.

Model	AUC	Accuracy	Precision	Recall	F1-Score
LightGBM	**0** **.** **917**	**0**.**810**	**0**.**857**	**0**.**667**	**0**.**750**
Logistic Regression	0.870	0.762	0.833	0.556	0.667
Random Forest	0.843	0.667	0.750	0.333	0.462
SVM	0.833	0.667	0.667	0.444	0.533
AdaBoost	0.824	0.762	0.833	0.556	0.667
KNN	0.796	0.667	0.750	0.333	0.462
Decision Tree	0.778	0.762	0.833	0.556	0.667
XGBoost	0.667	0.619	0.571	0.444	0.500

**Figure 3:**
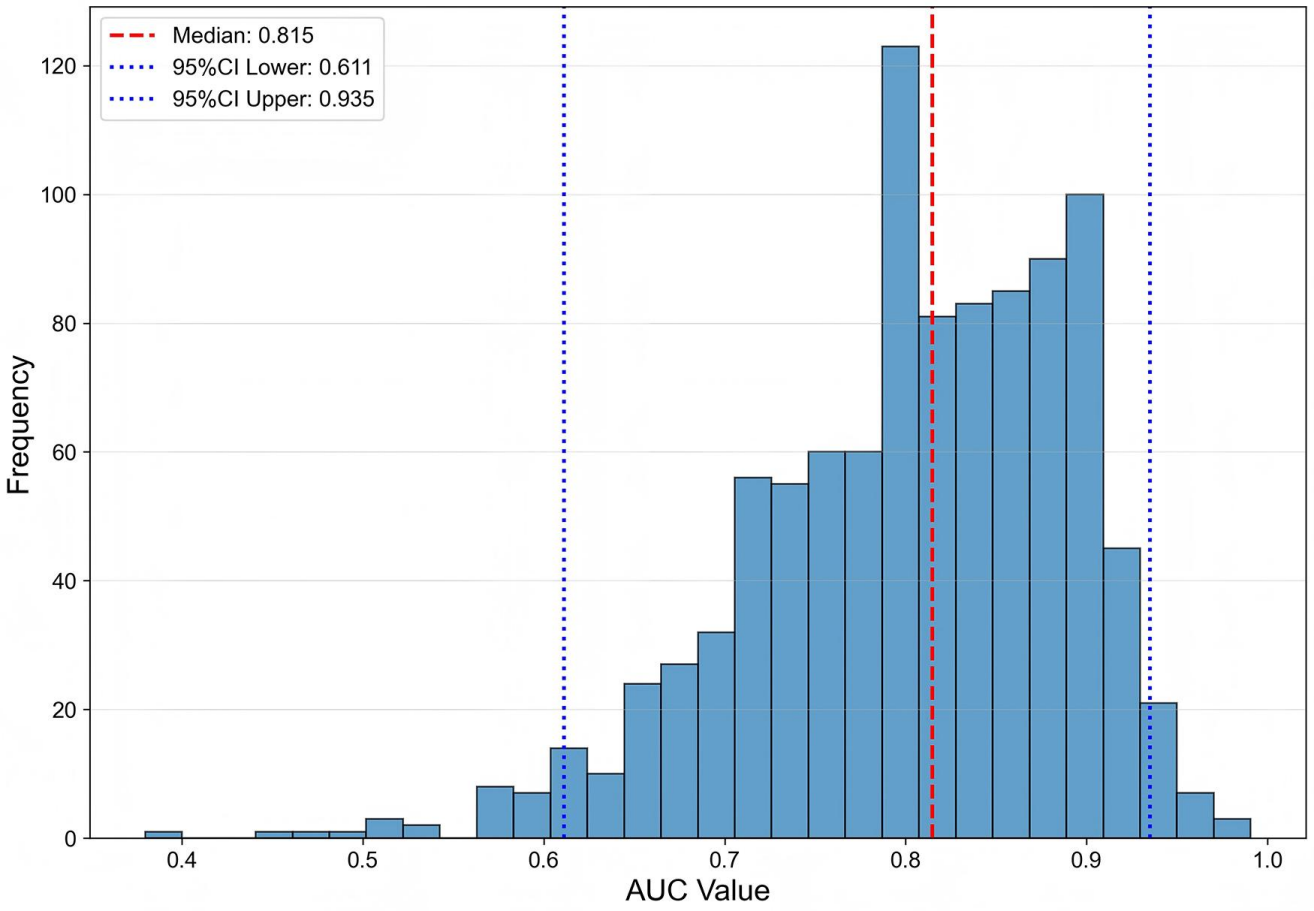
Distribution of AUC values from 1,000 bootstrap resamples for the LightGBM model in the PeAF group. This figure displays the distribution of AUC values obtained from 1,000 bootstrap resampling iterations. The box plot indicates the median AUC (0.815) and interquartile range, while the dashed lines represent the 95% confidence interval (0.611, 0.935).

To evaluate the potential variability in performance estimation due to the smaller sample size in the PeAF subgroup, we performed a bootstrap resampling analysis on the optimal LightGBM model for the PeAF group (Figure 3). Although the median AUC derived from bootstrap resampling was lower than the AUC obtained on the original test set (0.917), it provides a more conservative and potentially more realistic estimate of the model's generalizability. The results clearly demonstrate that despite the limited sample size in the PeAF cohort, the LightGBM model exhibited robust predictive performance. The range of performance estimates remained within an acceptable margin, and the majority of the AUC distribution was significantly above the random chance level (AUC = 0.5). This analysis enhances the reliability of the findings from this smaller subgroup study.

### 3.4 Decision curve analysis

While a model's high discriminative ability (e.g., AUC) is essential, it does not directly equate to clinical utility. To evaluate the predictive model's value in real-world clinical decision-making, we performed DCA. DCA quantifies the clinical usefulness of a model by calculating the net benefit obtained from using the model to guide clinical interventions, in comparison to default strategies of “Treat All” or “Treat None” patients, across a spectrum of threshold probabilities.

**Figure 4:**
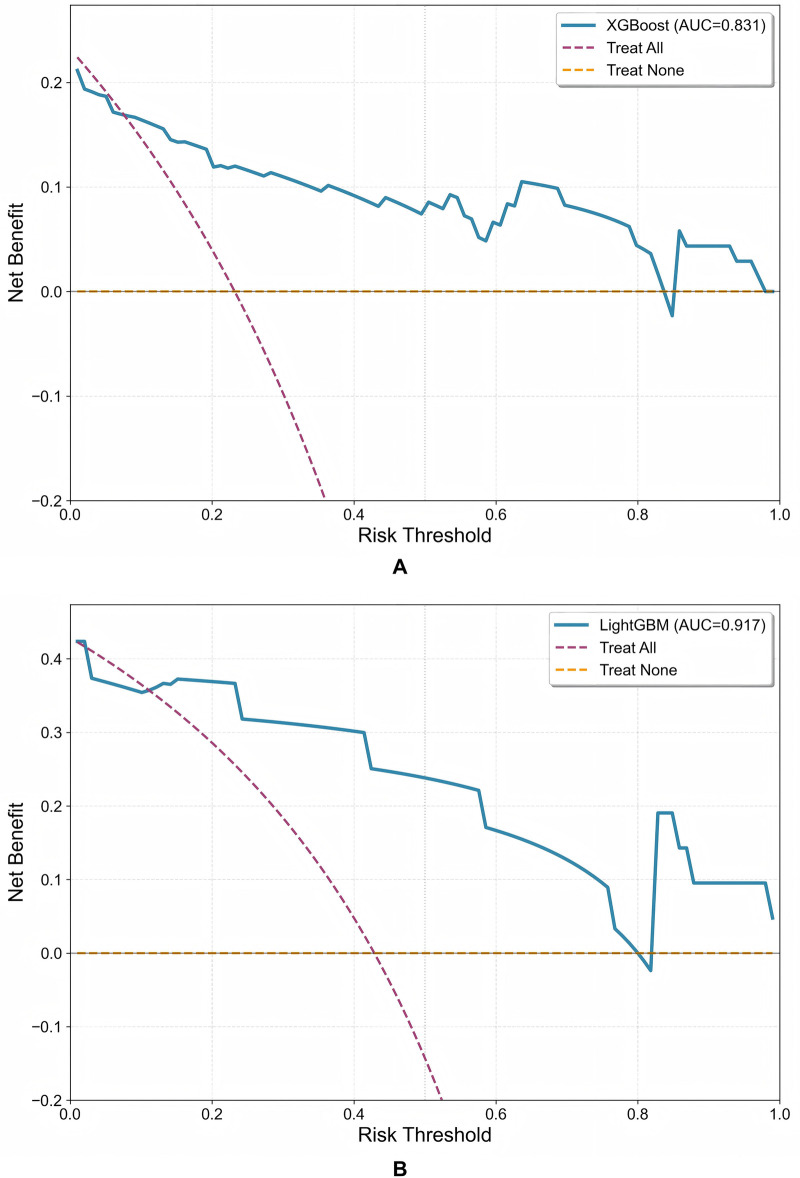
Decision curve analysis (DCA) of the predictive models. **(A)** PaAF group (XGBoost model); **(B)** PeAF group (LightGBM model). The *Y*-axis represents the clinical net benefit, and the *X*-axis represents the threshold probability. When the model's net benefit curve (blue) is above the reference lines for “Treat All” (purple) and “Treat None” (yellow), it indicates that using the model to guide clinical decision-making yields a positive net benefit within the corresponding probability range.

As shown in Figure 4, for the PaAF (Figure 4A), the clinical net benefit of the XGBoost model consistently remained above the reference lines for “Treat All” and “Treat None” across a wide threshold probability range of approximately 0.1–0.6.This indicates that within this decision range, using the model to stratify patients for targeted intervention can avoid overtreatment of low-risk patients while ensuring effective management of high-risk patients, thereby achieving a higher clinical net benefit. In the PeAF group (Figure 4B), the LightGBM model also demonstrated significant clinical utility. Its net benefit curve was above the two reference lines within the threshold probability range of 0.1–0.7. This result validates the model's effectiveness in identifying high-risk patients after PeAF ablation, suggesting that clinicians can formulate individualized follow-up or intervention strategies based on the recurrence probability output by the model (e.g., within the 10% to 70% range).

### 3.5 Model interpretation with SHAP

**Figure 5:**
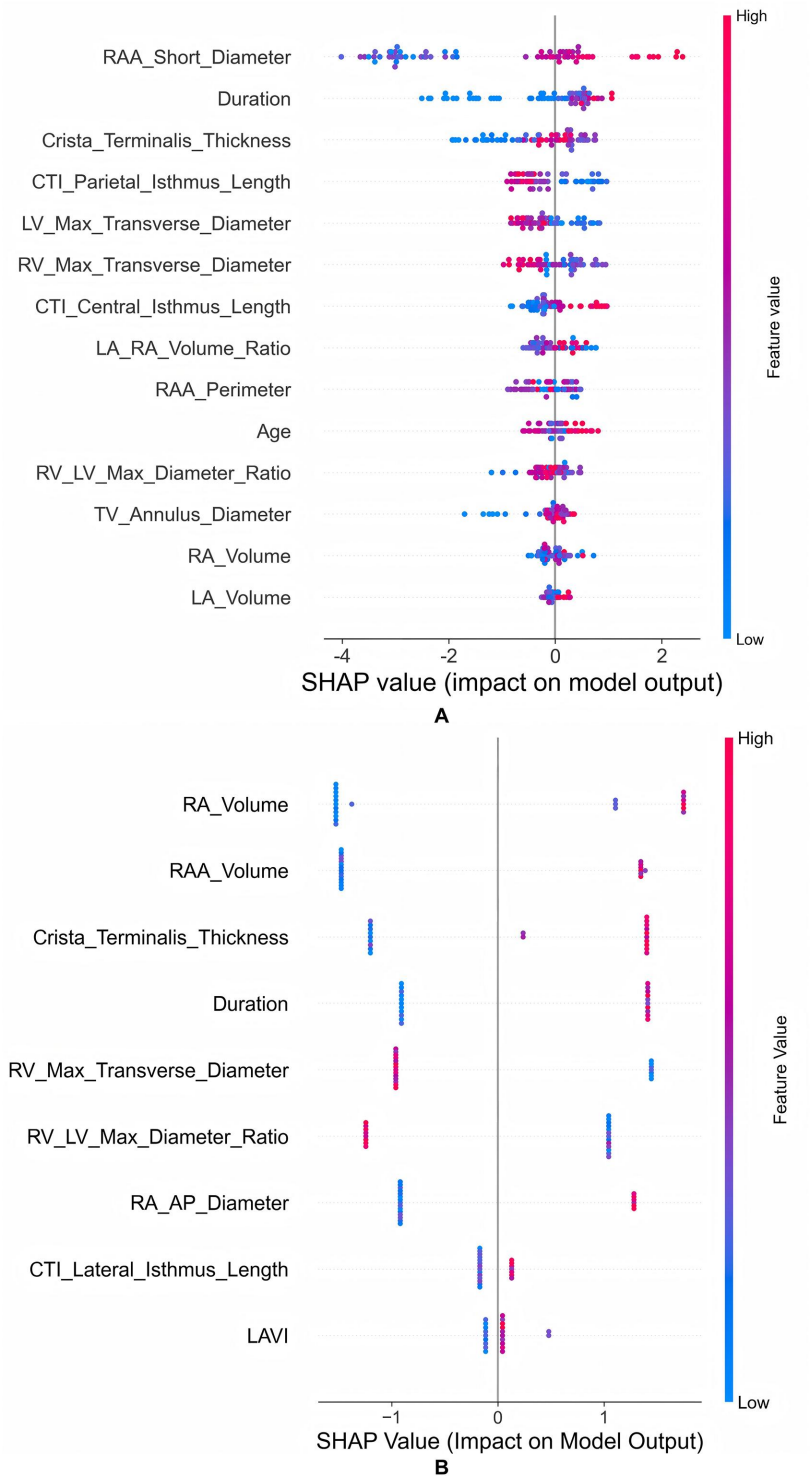
SHAP summary plots (beeswarm plots) for the optimal models predicting atrial fibrillation recurrence after ablation. **(A)** Results for the XGBoost model in the paroxysmal AF (PaAF) cohort. **(B)** Results for the LightGBM model in the persistent AF (PeAF) cohort. In each plot, each dot represents an individual patient. The position on the *x*-axis indicates the SHAP value (i.e., the feature's impact on the model's output towards higher recurrence risk), and the color represents the original feature value (red for high values, blue for low values). Features are ranked from top to bottom by their mean absolute SHAP value (global importance).

SHAP analysis was employed to reveal AF type-specific key predictors. For PaAF (Figure 5A), the RAA short diameter was the most important feature, with larger values (red dots) increasing the risk of recurrence (positive SHAP value). For PeAF (Figure 5B), RA volume and RAA volume were the top predictors, similarly associated with higher risk.

**Figure 6:**
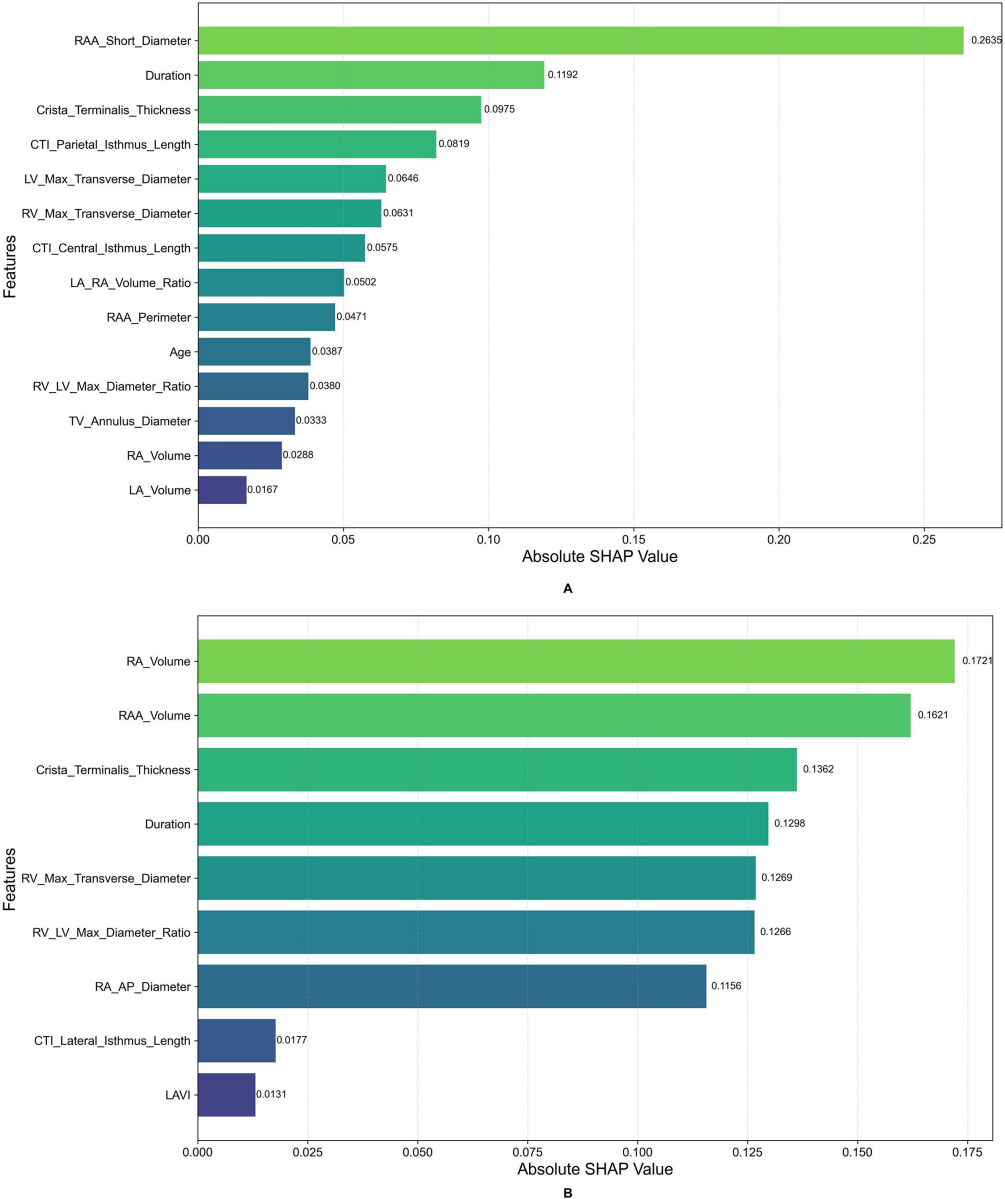
Global feature importance ranking based on SHAP values for the optimal models in different AF types. **(A)** The XGBoost model in the paroxysmal AF (PaAF) cohort. **(B)** The LightGBM model in the persistent AF (PeAF) cohort. In each bar plot, features are ranked vertically by their mean absolute SHAP value (horizontal axis), which quantifies each feature's overall contribution to the model's predictions. Bar color corresponds to the feature's original scale, aiding in visual distinction.

**Figure 7:**
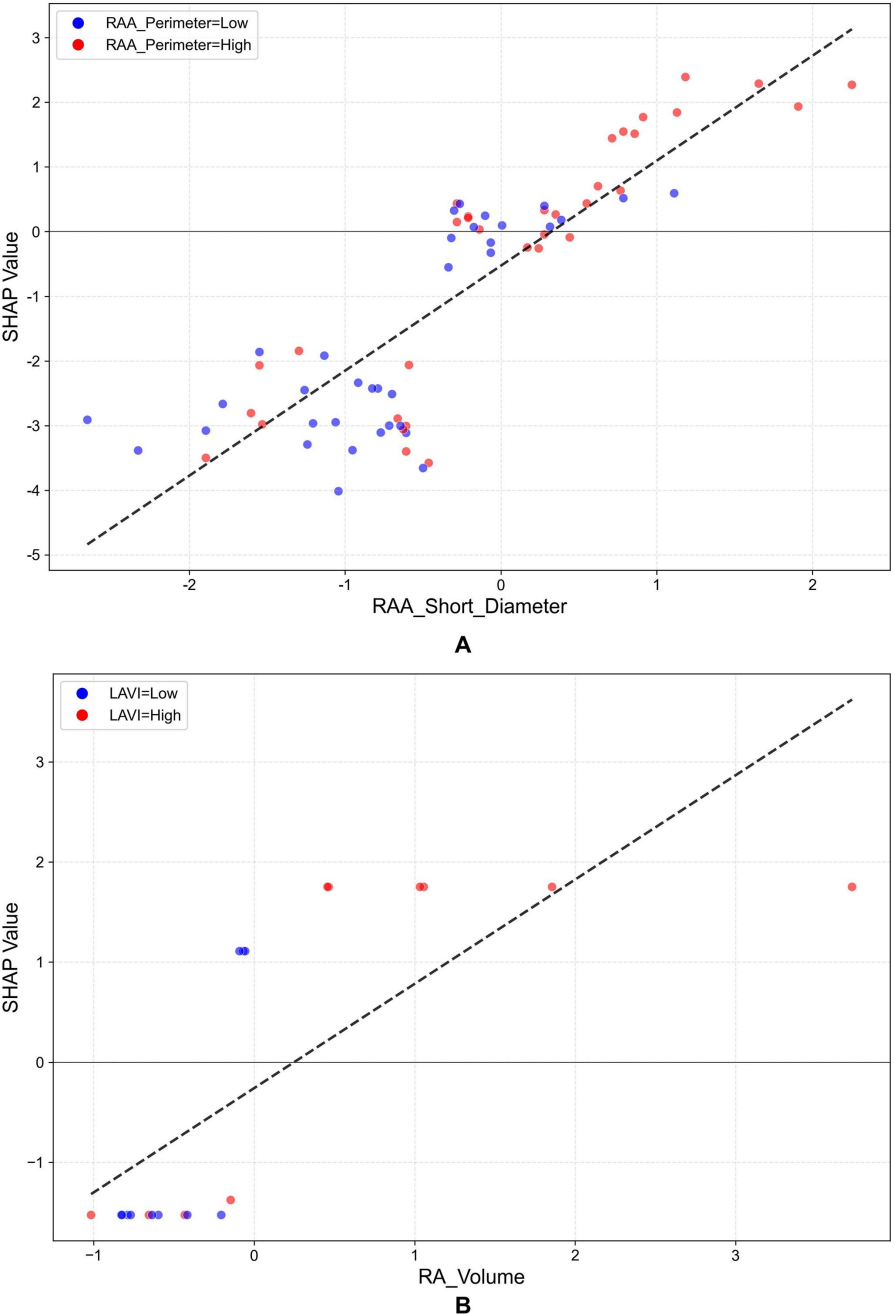
SHAP dependence plots for key predictors. **(A)** PaAF group: Relationship between the RAA short diameter and recurrence risk, where point color indicates the feature with the strongest interaction effect (RAA Perimeter); **(B)** PeAF group: Relationship between RA Volume and recurrence risk, where point color indicates the LAVI. This figure reveals the nonlinear relationships between key features and recurrence risk, as well as interaction effects between features.

Figure 6 provides a quantitative summary of feature importance. For PaAF (Figure 6A), the RAA Short Diameter (mean |SHAP| = 0.264) was the most important predictor. For PeAF (Figure 6B), the RA Volume (mean |SHAP| = 0.172) emerged as the top contributor. This figure provides a complementary, high-level overview of the key drivers for each model, distinct from the individual-level explanations in Figure 5. Furthermore, SHAP dependence plots (Figure 7) revealed complex nonlinear relationships and interaction effects between key predictors and the risk of recurrence. For PaAF, as shown in Figure 7A, the RAA short diameter demonstrates a positive correlation with recurrence risk, although its influence does not follow a simple linear progression. More importantly, the color of the data points in the plot (representing RAA perimeter) indicates a significant interaction effect. When the RAA short diameter falls within a medium range, the risk-promoting effect is more pronounced if it is accompanied by a larger RAA perimeter (red points). This interaction between the RAA short diameter and perimeter suggests that clinical assessment should consider the sphericity index of the right atrial appendage rather than relying on a single linear measurement. For PeAF, the SHAP dependence plot (Figure 7B) elucidates a complex, non-linear relationship between atrial remodeling and recurrence risk. It confirms that RA volume is a core driver (main effect) of the model's prediction, showing a monotonically positive association with higher SHAP values. Critically, the analysis reveals a significant effect modification by the LAVI. Among patients with comparable RA volumes, those exhibiting a concomitantly higher LAVI (red points) demonstrate a markedly accelerated increase in predicted risk (SHAP values) compared to their counterparts with a lower LAVI (blue points). This pattern strongly suggests a positive statistical interaction between RA volume and LAVI, indicating that the detrimental impact of RA enlargement is substantially potentiated by coexistent LA dilation.The mechanistic implication is that PeAF recurrence may be propelled not by isolated unilateral atrial enlargement, but rather by a coupled, synergistic process of biatrial remodeling. The identification of this “biatrial dilation phenotype” may delineate a distinct patient subgroup with more advanced, diffuse atrial myopathy.

**Figure 8:**
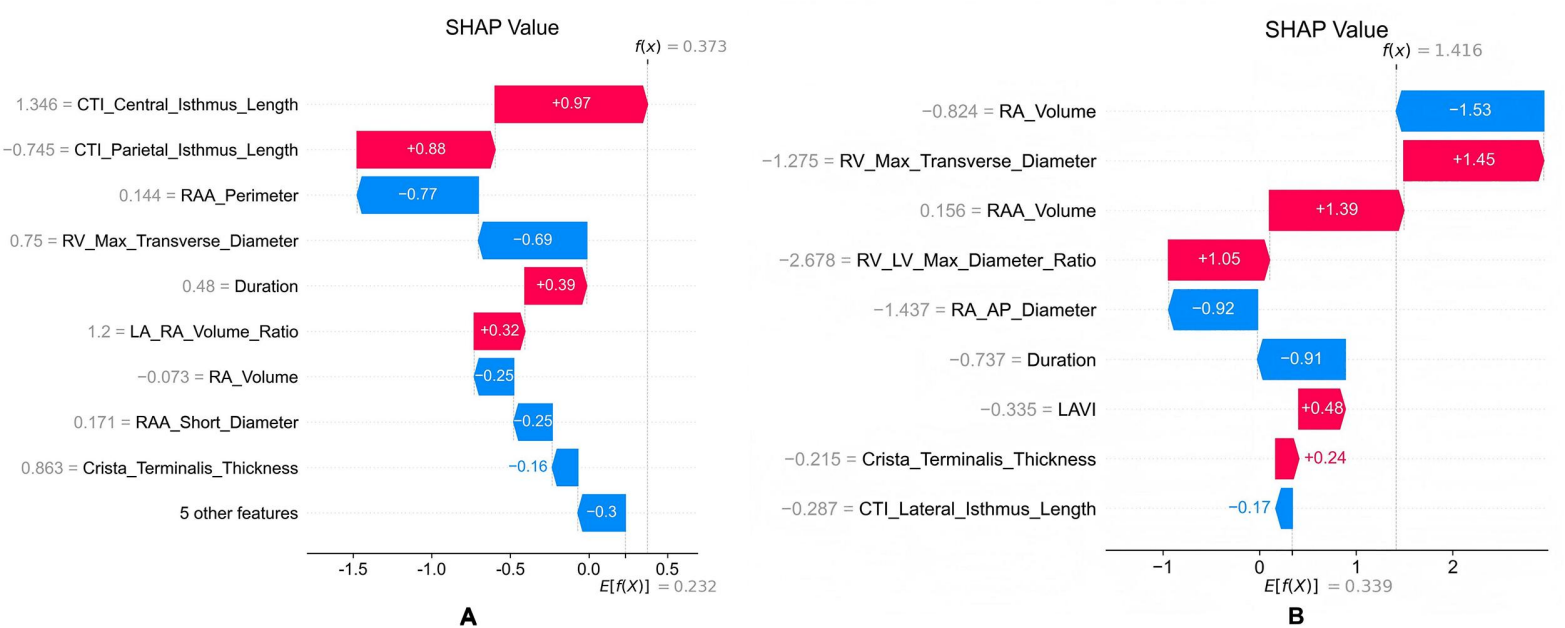
SHAP local explanation waterfall plots for individualized recurrence risk prediction. **(A)** PaAF group example: a patient who did not experience actual recurrence but had a model-predicted probability near the decision threshold (0.502); **(B)** PeAF group example: a patient with actual recurrence (predicted probability 0.578). Starting from the average predicted value for all patients, the chart sequentially illustrates how each feature value drives the final prediction towards the model's output. Red bars indicate that the feature increases the recurrence risk, while blue bars indicate a decreased risk.

SHAP waterfall plots provide an individualized decomposition of recurrence risk, clearly demonstrating the ability of various features to exert opposing effects, thereby offering a basis for precise risk assessment. As shown in Figure 8A, which illustrates a patient with PaAF who did not experience recurrence despite a model-predicted probability close to the decision threshold (0.502). Although features such as the central isthmus length of the CTI elevated the risk, other features, notably a larger RAA perimeter, exerted a significant protective effect, offsetting the high-risk factors. Figure 8B, corresponds to a patient with PeAF who experienced recurrence. Interestingly, most of this patient's cardiac structural parameters (e.g., right ventricular transverse diameter) indicated a protective effect. However, these anatomical advantages were completely offset by a few strong, unidentified risk factors (with SHAP values of +1.39 and +1.05), leading to a final high-risk prediction (0.578). This strongly suggests that for patients with PeAF, non-structural electrophysiological or molecular factors (such as the extent of fibrosis or ion channel function) may play a decisive role in recurrence. This finding alerts clinicians to look beyond imaging-based structural assessment and points towards future research directions, highlighting that integrating multimodal data is key to further improving prediction accuracy.

### 3.6 Web-based interactive prediction system and pilot testing

**Figure 9:**
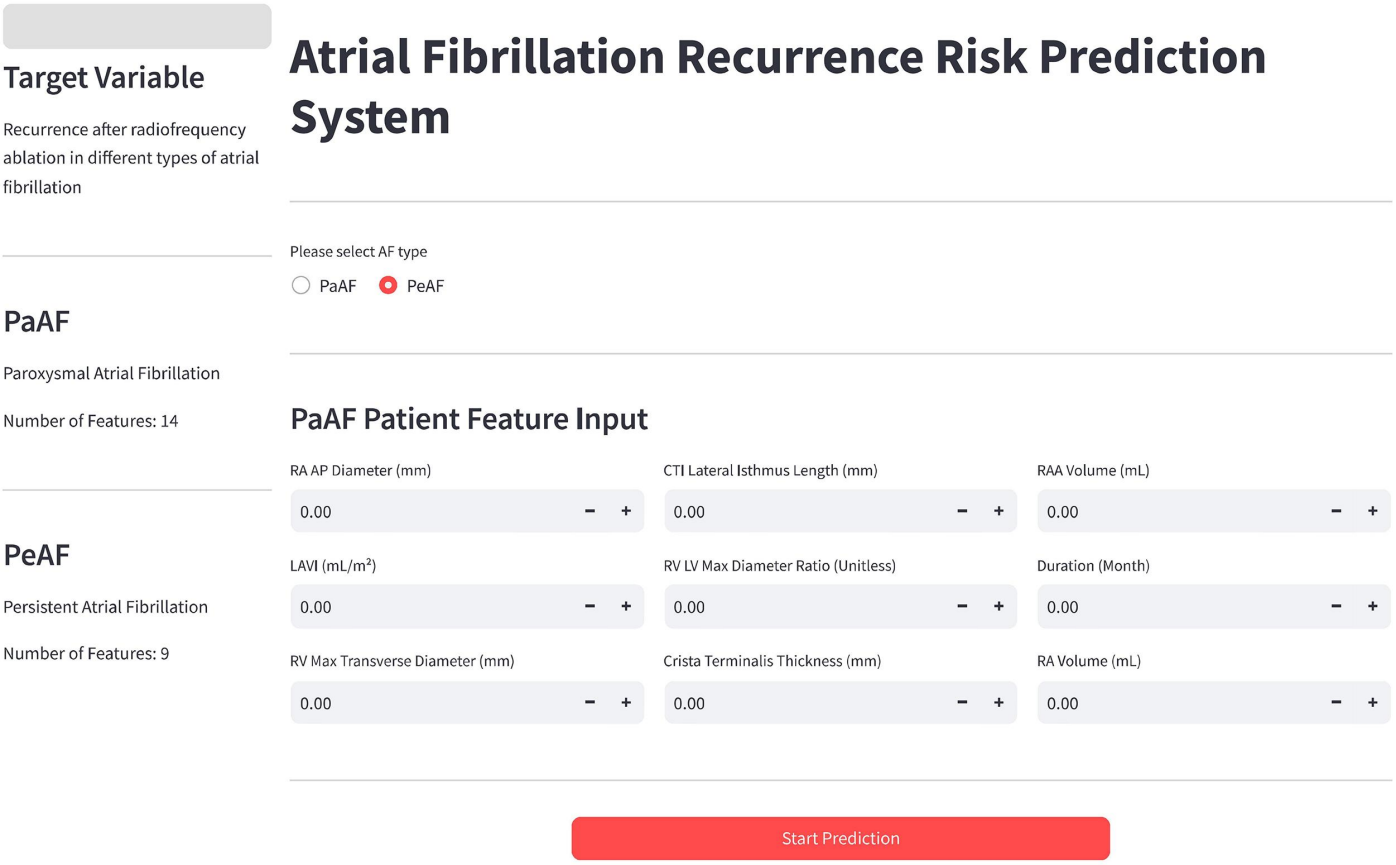
Interface of the interactive clinical decision support web tool developed based on the optimal machine learning models. This tool allows clinicians to select the atrial fibrillation type (PaAF or PeAF) and input patient characteristics to rapidly calculate the recurrence probability and visualize the SHAP feature importance ranking, providing transparent decision support for individualized prognosis assessment.

To facilitate clinical translation, we developed a web-based interactive tool based implementing the optimal models. This tool provides a user-friendly interface for clinicians to select the AF type (PaAF or PeAF), input patient characteristics, and instantly obtain a predicted recurrence risk probability (Figure 9). It concurrently generates a SHAP-based feature importance summary, delivering efficient and interpretable decision support. Currently, this system is undergoing a pilot implementation study at three tertiary hospitals. The primary objectives of this pilot include: (1) collecting feedback from clinicians on the system's interface and operational workflow; (2) observing how the tool embeds into existing outpatient assessment workflows; and (3) conducting a preliminary validation of its predictive accuracy in a small, prospectively enrolled consecutive patient cohort (planned *n* = 50). This pilot is designed to gather initial evidence on the tool's real-world practicality and performance, informing further optimization and paving the way for a larger-scale, multi-center effectiveness study.

## 4 Discussion

This study developed distinct machine learning models to predict recurrence after catheter ablation for PaAF and PeAF, accounting for their differences in pathological substrate. The results demonstrated that the XGBoost (for PaAF) and LightGBM (for PeAF) models exhibited excellent predictive performance, with AUCs of 0.831 and 0.917, respectively. More importantly, through SHAP explainable analysis, we systematically identified and quantified the pivotal role and type-specificity of right atrial structural parameters: the RAA short diameter was the most important predictor for PaAF recurrence, while the overall RA volume contributed most to predicting PeAF recurrence. This study not only provides accurate prediction tools but also transforms ML models from “black-box” predictors into “discovery tools” capable of revealing potential AF recurrence mechanisms.

### 4.1 Association between right atrial structural parameters and AF recurrence post-ablation

The primary contribution of this study lies in transcending the traditional “left-atrium-centric” paradigm and providing robust evidence that right atrial structural parameters are independent and powerful predictors of AF recurrence after ablation. The AF type-specific predictors revealed by SHAP analysis may profoundly reflect the distinct pathophysiological mechanisms underlying PaAF and PeAF.

1. **PaAF and the Right Atrial Appendage:** In PaAF, the RAA short diameter emerged as the most critical predictor, likely related to its role as a significant origin site for non-pulmonary vein triggers. The unique trabeculated structure of the RAA is prone to forming slow conduction and micro-reentry. An increased short diameter may reflect local abnormalities in muscle bundle arrangement, fibrosis, or dilation, thereby creating an electrophysiological substrate more susceptible to sustained focal activity. This aligns with findings by Pan et al. (18) and is further supported by the SHAP dependence plot, which revealed an interaction effect between the RAA short diameter and perimeter (Figure 6). This indicates that for PaAF patients, assessing local structural changes in the RAA is crucial for identifying high recurrence risk.
2. **PeAF and the Right Atrial Volume:** For PeAF, the global RA volume superseded local parameters as the primary predictor, which is more consistent with the nature of PeAF—more extensive and diffuse biatrial electrical and structural remodeling. Chronic atrial overload and cumulative fibrosis lead to overall atrial enlargement, and an increased RA volume is a macroscopic manifestation of this progressive atrial myopathy. The study by Izumi et al. (25) indicated that AF duration is a key factor in right heart structural remodeling, providing a rationale for the significantly larger RA volume observed in our PeAF patients (who typically have a longer disease duration). Therefore, RA volume may serve as a surrogate marker for the severity of atrial myopathy in PeAF patients, indicating a more refractory substrate that is more likely to recur post-ablation. More importantly, SHAP analysis revealed a significant synergistic interaction effect between RA volume and the left atrial volume index (LAVI) (Figure 7B). This strongly suggests that PeAF recurrence is not driven by isolated atrial enlargement, but rather by a coupled, synergistic process of biatrial remodeling, as marked by the biatrial dilation phenotype.

In summary, the findings of this study, together with recent evidence emphasizing the role of the right atrium (26, 27), advance the understanding of AF recurrence mechanisms, promoting a paradigm shift from focusing solely on the left atrium towards a comprehensive assessment of biatrial interactions. The study by Boyuk et al. (28) on the superior predictive value of combined biatrial indices over single ones also reinforces the importance of the “biatrial consideration” concept revealed by our model.

### 4.2 Model performance and advantages of machine learning algorithms

Currently, machine learning approaches are increasingly being employed to predict the risk of atrial fibrillation (AF) recurrence (29–33). Notably, the excellent performance of our models [AUC: 0.831(PaAF), 0.917(PeAF)] aligns with the high predictive accuracy demonstrated in recent ML studies on AF outcomes, which frequently report AUCs above 0.85 (34, 35). This superior discriminative ability can be attributed to two pivotal and novel design elements of our work. First, the models were explicitly constructed by distinguishing between PaAF and PeAF, establishing specific predictive models tailored to the electrophysiological and structural remodeling differences between AF types; second, this study systematically incorporated right atrial structural parameters, breaking through the limitation of traditional models that focus primarily on left atrial indicators, thus providing a more comprehensive reflection of the synergistic role of biatrial remodeling in AF recurrence.

These results are highly consistent with trends in the application of machine learning in cardiovascular outcome prediction. Ensemble learning algorithms, such as XGBoost and LightGBM, are particularly effective at capturing complex nonlinear relationships and interaction effects among multi-dimensional clinical features (32, 33, 36, 37). For instance, Nie et al. (32) demonstrated the effectiveness of XGBoost after integrating multifaceted clinical data, and Budzianowski et al. (33) and Guan et al. (36) highlighted the significant value of such algorithms in enhancing predictive accuracy. Extending this evidence, Askarinejad et al. (37) also reported outstanding performance (accuracy: 92.5%, AUC: 0.96) using CatBoost, another powerful ensemble algorithm, for predicting post-ablation AF recurrence. Building upon these foundations, our study demonstrates that by incorporating right atrial parameters and stratifying by AF type, such ensemble methods can more fully leverage key predictors, thereby further optimizing discriminative ability within this specific pathophysiological context.

However, a deeper comparison reveals a pivotal divergence: the identity of the most important predictors differs substantially across studies. While our SHAP analysis identified right atrial (RA) parameters—RAA short diameter for PaAF and RA volume for PeAF—as paramount, other high-performance models have emphasized different feature sets, such as left atrial/pulmonary vein features (30), clinical composites (e.g., renal function) (32), or post-ablation scar characteristics (29). The apparent discrepancy in feature importance between our study and other high-performing models is not a contradiction but a meaningful reflection of key methodological and biological principles. This variation arises from prediction-target specificity (where the atrial substrate itself is paramount for recurrence prediction, unlike other endpoints like stroke) (38), our deliberate cohort stratification by AF type (which preserved distinct PaAF- and PeAF-specific signals, such as RAA morphology vs. global RA volume, that would be diluted in a combined model), and the fundamental constraint of feature space definition (our hypothesis-driven, comprehensive quantification of the right atrium enabled our models to identify its importance, whereas many prior studies did not include an equally rich representation of this chamber). Therefore, the comparison underscores that our findings—the critical role of the right atrium—are most clearly revealed through the synergistic approach of AF-type-specific modeling coupled with dedicated, comprehensive biatrial phenotyping.

Another highlight of this study is the combination of high-performance prediction with high-transparency explanation. Similar to the aforementioned studies, we utilized the SHAP framework to demystify the “black box”. However, we went a step further by quantifying the AF type-specificity of predictor importance rankings. This provides clinicians with insights beyond traditional statistical models: it not only tells physicians “which factors are important” but also clearly indicates “which factors are most important for different types of patients”. This kind of machine learning-based “data-driven subgroup analysis” offers a novel quantitative academic perspective for understanding the heterogeneity of AF.

### 4.3 Strengths and limitations

The strengths of this study include: (1) constructing separate machine learning prediction models based on the pathological heterogeneity of PaAF and PeAF; (2) systematically exploring the predictive value of right atrial parameters, promoting a paradigm shift from a “left-atrium-centric” view to a “biatrial consideration” approach; and (3) developing an interactive web tool integrated with SHAP explanations, directly facilitating clinical translation.

However, this study also has several limitations. The primary limitation of this study is the relatively small sample size in the PeAF subgroup (*n* = 67), which increases the risk of model overfitting and may lead to an optimistic estimation of performance on the internal test set. Although bootstrap resampling was employed to quantify the variability and provide a more conservative performance estimate (median AUC: 0.815, 95% CI: 0.611–0.935), the wide confidence interval reflects this inherent instability. Therefore, the results related to the PeAF model, while promising, should be interpreted with caution and require further validation in larger, prospective cohorts. Second, there is potential bias in recurrence monitoring. AF recurrence was primarily detected through symptom-driven reporting and scheduled follow-up ECGs, potentially missing asymptomatic recurrences. The limited sensitivity of this monitoring protocol may lead to an underestimation of the true recurrence rate and could affect the model in two ways: on one hand, it might inflate the model's discriminative ability (AUC) in internal validation; on the other hand, it might lead to an underestimation of the association between certain risk factors (e.g., right atrial parameters) and true recurrence. Finally, the current model is primarily based on clinical and CT imaging features. Future integration of multimodal data, such as intracardiac electrograms (e.g., voltage mapping), blood biomarkers (e.g., B-type natriuretic peptide, fibrosis markers), and even genetic information, holds promise for building more comprehensive and powerful predictive models. Despite these limitations, this study provides a valuable foundation for the precise prognosis assessment of AF.

## 5 Conclusion and future perspectives

In conclusion, this study confirms that machine learning-based predictive models can more accurately predict recurrence after atrial fibrillation ablation and, for the first time, establishes right atrial structural parameters as core predictors. Combined with the transparent decision explanation provided by SHAP, this study offers a novel and reliable tool for precise prognostic assessment of AF. Future research directions should include external validation with expanded sample sizes, integration of multimodal data such as intracardiac electrophysiological parameters and blood biomarkers to build more powerful predictive models, and exploration of integrating the web tool into clinical workflows for real-time risk assessment, ultimately achieving individualized holistic management for AF patients.

## Data Availability

The raw data supporting the conclusions of this article will be made available by the authors, without undue reservation.
