# Supplementary material for: Atrial fibrillation type-specific prediction of recurrence after catheter ablation: the pivotal role of right atrial remodeling revealed by explainable machine learning
Source: Front Cardiovasc Med. 2026 Apr 29;13:1805262. doi: 10.3389/fcvm.2026.1805262 (PMC13167954; doi:10.3389/fcvm.2026.1805262)
Supplement: Supplementary file 1 [file Table1.docx]

Supplementary Material

**Supplementary Table S1.** Detailed Process of Feature Selection for PaAF: Results from Boruta and RFECV.

| Feature | Boruta Selection  Selection | RFECV Selection  Selection  Values | Is in the intersection |
| --- | --- | --- | --- |
| RA_Volume | True | True | True |
| LA_Volume | True | True | True |
| LA_RA_Volume_Ratio | True | True | True |
| RAA_Morphology | False | False | False |
| RAA_Short_Diameter | True | True | True |
| RAA_Perimeter | True | True | True |
| RAA_Anatomical_Spread_Angle | False | True | False |
| RA_AP_Diameter | False | True | False |
| TV_Annulus_Diameter | True | True | True |
| RV_Max_Transverse_Diameter | True | True | True |
| LV_Max_Transverse_Diameter | True | True | True |
| RV_LV_Max_Diameter_Ratio | True | True | True |
| Crista_Terminalis_Thickness | True | True | True |
| CTI_Parietal_Isthmus_Length | True | True | True |
| CTI_Central_Isthmus_Length | True | True | True |
| BSA | False | True | False |
| Diabetes | False | False | False |
| Heart_Failure | False | False | False |
| CHA2DS2-VASc Score | False | False | False |
| Duration | True | True | True |
| Hypertension | False | False | False |
| CHD | False | False | False |
| Cerebral_Infarction_TIA | False | False | False |
| Hyperlipidemia | False | False | False |
| BMI | False | True | False |
| Gender | False | False | False |
| Age | True | True | True |
