# Supplementary material for: Atrial fibrillation type-specific prediction of recurrence after catheter ablation: the pivotal role of right atrial remodeling revealed by explainable machine learning
Source: Front Cardiovasc Med. 2026 Apr 29;13:1805262. doi: 10.3389/fcvm.2026.1805262 (PMC13167954; doi:10.3389/fcvm.2026.1805262)
Supplement: Supplementary file 2 [file Table2.docx]

Supplementary Material

**Supplementary Table S2.** Detailed Process of Feature Selection for PeAF: Results from Boruta and RFECV.

| Feature | Boruta Selection  Selection | RFECV Selection  Selection  Values | Is in the intersection |
| --- | --- | --- | --- |
| RAA_Volume | True | True | True |
| RA_Volume | True | True | True |
| LA_RA_Volume_Ratio | False | False | False |
| RAA_Length | False | False | False |
| RAA_Anatomical_Spread_Distance | False | False | False |
| RA_AP_Diameter | True | True | True |
| TV_Annulus_Diameter | False | False | False |
| RV_Max_Transverse_Diameter | True | True | True |
| LV_Max_Transverse_Diameter | False | False | False |
| RV_LV_Max_Diameter_Ratio | True | True | True |
| Crista_Terminalis_Thickness | True | True | True |
| CTI_Parietal_Isthmus_Length | True | False | False |
| CTI_Lateral_Isthmus_Length | True | True | True |
| BSA | False | False | False |
| Duration | True | True | True |
| LAVI | True | True | True |
| Hypertension | False | False | False |
| BMI | False | False | False |
| Age | False | True | False |
