# Supplementary material for: Atrial fibrillation type-specific prediction of recurrence after catheter ablation: the pivotal role of right atrial remodeling revealed by explainable machine learning
Source: Front Cardiovasc Med. 2026 Apr 29;13:1805262. doi: 10.3389/fcvm.2026.1805262 (PMC13167954; doi:10.3389/fcvm.2026.1805262)
Supplement: Supplementary file 4 [file Table4.docx]

Supplementary Material

**Supplementary Table S4.** Optimal hyperparameters for the eight machine learning models in the persistent atrial fibrillation (PeAF) cohort. The hyperparameter search was conducted via grid search, and the final combination was selected based on the highest mean AUC achieved during 5-fold cross-validation on the training set.

| Algorithm | Hyperparameter | Search Space/Candidate Values | Optimal Parameters |
| --- | --- | --- | --- |
| XGBoost​ | max_depth | [3, 5, 7, 10] | 3 |
|  | learning_rate | [0.01, 0.1, 0.2, 0.3] | 0.1 |
|  | n_estimators | [50, 100, 200] | 50 |
| LightGBM​ | learning_rate | [0.2, 0.3, 0.4] | 0.2 |
|  | n_estimators | [120, 150, 200, 250] | 200 |
|  | max_depth | [10, 12] | 10 |
| Random Forest | max_depth | [None, 10, 15, 20] | None |
|  | n_estimators | [50, 100, 200] | 200 |
|  | min_samples_split | [2, 5, 10] | 2 |
| Support Vector Machine (SVM)​ | C | [0.1, 1, 2, 5] | 1 |
|  | gamma | ['scale', 'auto'] | scale |
|  | kernel | ['linear', 'rbf'] | rbf |
| K-Nearest Neighbors (KNN)​ | n_neighbors | [3, 5, 7, 9] | 7 |
|  | weights | ['uniform', 'distance'] | distance |
|  | p | [1, 2] | 1 |
| Logistic Regression​ | C | [0.01, 0.1, 0.5, 1, 2] | 0.1 |
|  | penalty | ['l1', 'l2'] | 12 |
|  | solver | ['liblinear', 'saga'] | liblinear |
| Decision Tree | max_depth | [None, 5, 10, 15] | None |
|  | min_samples_split | [5, 8, 10] | 10 |
|  | min_samples_leaf | [3, 4, 5] | 3 |
| AdaBoost | n_estimators | [50, 100, 200] | 100 |
|  | learning_rate | [0.01, 0.1, 1] | 1 |
